# Comparative study between two recombinant human NPH insulin formulations for the treatment of type 2 diabetes mellitus

**DOI:** 10.1590/2359-3997000000140

**Published:** 2016-01-01

**Authors:** Nelson Rassi, Sandra Maria Campos Teixeira de Moraes, Adriana Ganam Alves, Daniela Cunha Cavalheiro, Josianny Mesquita Moreira, Karini Bruno Bellório, Fernanda Cruvinel de Abreu, Paulo Roberto Mendonça Prata, Leonardo de Souza Teixeira, Salvador Rassi

**Affiliations:** 1 Hospital Geral de Goiânia Alberto Rassi Goiânia GO Brasil Hospital Geral de Goiânia Alberto Rassi, Goiânia, GO, Brasil; 2 Universidade Federal de Goiás (UFG) Goiânia GO Brasil Universidade Federal de Goiás (UFG), Goiânia, GO, Brasil; 3 Instituto de Ciências Farmacêuticas (ICF) Goiânia GO Brasil Instituto de Ciências Farmacêuticas (ICF), Goiânia, GO, Brasil

**Keywords:** Comparative study, NPH insulin, type 2 diabetes mellitus, glycated hemoglobin A, public health

## Abstract

**Objective:**

To compare the effects of the neutral protamine Hagedorn (NPH) recombinant human insulin formulations Gansulin and Humulin N^®^ on the glycemic control of patients with type 2 diabetes mellitus (T2DM).

**Subjects and methods:**

Prospective, double-blind, randomized, parallel, single-center study of 37 individuals with T2DM treated with NPH insulin formulations. The Tukey-Kramer test for multiple comparisons, the Wilcoxon paired comparison test and the Chi-Square test were used for the statistical analyses. The significance level was set at 5% (p < 0.05).

**Results:**

The NPH insulin formulations Humulin and Gansulin similarly reduced the HbA1c levels observed at the end of the study compared with the values obtained at the beginning of the study. In the Humulin group, the initial HbA1c value of 7.91% was reduced to 6.56% (p < 0.001), whereas in the Gansulin group, the reduction was from 8.18% to 6.65% (p < 0.001). At the end of the study, there was no significant difference between the levels of glycated hemoglobin (p = 0.2410), fasting plasma glucose (FG; p = 0.9257) and bedtime plasma glucose (BG; p = 0.3906) between the two insulin formulations. There was no nt difference in the number of hypoglycemic events between the two insulin formulations, and no severe hyp episodes were recorded.

**Conclusion:**

This study demonstrated similar glycemic control by NPH insulin Gansulin compared with human insulin Humulin N^®^ in patients with T2DM.

## INTRODUCTION

Projections by the World Health Organization (WHO) indicate that the prevalence of diabetes worldwide will exceed 300 million in 12 years and that developing countries will contribute significantly to these numbers (1,2). Diabetes mellitus (DM) exerts a high economic burden on both individuals and society. Such costs are associated not only with disease treatment and complications but also with expenses involving sick leave, absenteeism and early retirement (3). The costs incurred by patients with diabetes are up to three times higher than the costs incurred by individuals not affected by this disease, constituting a challenge for public health agencies (4,5).

A prospective and comparative drug therapy follow-up study of patients with type 2 diabetes mellitus (T2DM) conducted in community health centers demonstrated that a 1% reduction in HbA1c levels can be implemented with an mean annual investment of R$ 456.05 per patient, which includes medications and glucose monitoring reagent strips (6). This information indicates that T2DM treatment represents a high economic burden for government expenditure and that measures to reduce these expenses are necessary.

The use of neutral protamine Hagedorn (NPH) insulin contributes significantly to glycemic control (7,8). Assunção and cols. found that 41% of patients were not using the recommended dose of oral antidiabetic drugs, which likely led them to require insulin therapy earlier (9). Compared with the insulin analogs glargine and detemir, which are more expensive, NPH insulin has a similar efficacy; however, more nocturnal hypoglycemic events are observed with its use (7,10-12).

Currently, NPH insulin is produced by only two pharmaceutical companies, one American and one Danish, which produce the insulin formulations Humulin N and Novolin N, respectively. Two other companies have a small participation in this lucrative healthcare market: Indar (Ukraine) and Dongbao (China). Until 2002, Brazil was part of this select group of insulin-producing countries, represented by Biobras, which was responsible for 80% of the insulin sales in the country. Biobras was sold to a Danish multinational and ceased the local production of the hormone, leaving Brazil dependent on imported insulin.

There are only two companies in the Brazilian pharmaceutical market that produce recombinant human NPH insulin, which then limits the price reduction in the competitive bidding process for the purchase of insulin by the Brazilian Ministry of Health. The availability of other types of NPH insulin in the national drug market could in theory make it accessible through reduced prices to the private and public health care systems.

Thus, the aim of this study was to compare the glycemic-controlling effects of a new human recombinant NPH insulin produced in China (Gansulin N) with an insulin that has been available in the Brazilian pharmaceutical market for decades (Humulin N).

## SUBJECTS AND METHODS

This prospective, double-blind, randomized, parallel, single-center study was approved by the Human and Animal Research Ethics Committee of Goiania General Hospital (*Hospital Geral de Goiânia*). Forty patients of both genders (9 females and 11 males for the randomized Humulin group; 10 females and 10 males for the randomized Gansulin group), aged 18 to 60 years and diagnosed with T2DM for over a year, according to the criteria of the Brazilian Diabetes Society (*Sociedade Brasileira de Diabetes*), were included in the study. The inclusion criteria for the study were the use of NPH insulin for more than 12 months, a body mass index (BMI) between 20 and 35 kg/m^2^ and glycated hemoglobin (HbA1c) levels between 7.5 and 10% at the time of selection. In addition, the patients had to agree to participate in the study and sign an informed consent form.

There were 18 consults during the clinical phase of the study, with one consult per week. The first three consults (first 3 weeks) corresponded to the phase of insulin dose adjustment, or phase 1, and preceded the randomization of patients. During this phase, the insulin doses were adjusted to achieve optimal glycemic control and homogeneity of the study population. The NPH insulin Humulin N^®^ manufactured by Eli Lilly and Company was administered to volunteers during phase 1.

A standard (step-by-step) relationship was not established to adjust the insulin dose. The investigator (a physician) was free to adjust the insulin dose, aiming to achieve plasma glucose levels established by the protocol.

The doses of insulin were adjusted weekly by the investigator, taking into account the mean plasma glucose levels observed during the previous 3 days. The adjustment of the insulin dose administered at bedtime was based on the mean plasma glucose levels obtained before breakfast (fasting glucose, FG), aiming to achieve a plasma glucose level between 70 and 100 mg/dL; the insulin dose administered before breakfast was based on the mean plasma glucose levels obtained at bedtime (bedtime glucose, BG), aiming to achieve a plasma glucose level between 70 and 120 mg/dL. For the double-blind study, drugs were placed in identical carton boxes so that neither physician nor volunteer could distinguish them. Only the statistician had access to the randomization list.

On the fourth consult, the study population was randomly divided into two groups: one group remained under treatment with NPH insulin Humulin N (Group 1 or Reference), and the other group had their insulin replaced by Gansulin N immediately after phase 1 (Group 2 or Test). Both the test and reference insulin formulations were administered once and/or twice daily (before breakfast and/or at bedtime) in combination with metformin at a minimum daily dose of 500 mg.

Plasma glucose concentrations were determined by the glucose dehydrogenase method, whereas HbA1c levels were measured by high performance liquid chromatography (HPLC). Creatinine levels were determined by the automated kinetic method, and C-peptide levels were measured by chemiluminescence.

Patients were instructed to perform self-monitoring of their capillary blood glucose levels at home whenever they suspected hypoglycemia (capillary blood glucose ≤ 70 mg/dL) and to treat the hypoglycemia.

The Tukey-Kramer test for multiple comparisons was used to analyze the general information of the groups for the factor insulin. The Wilcoxon paired comparison test was used to test the effect of the insulin formulations on the glucose profile, and the likelihood ratio Chi-square test was used to analyze hypoglycemic events. The significance level was set at 5% (p < 0.05). Three male patients, 2 from the Gansulin group and 1 from the control group, were excluded from the study at consults C13, C18 and C20 due to incorrect use of insulin and were not included in the statistical analyses.

## RESULTS

Table 1 lists the main variables (plasma glucose and C-peptide profile) of the 40 patients at the beginning of the study. The patients presented inadequate mean plasma glucose levels (FG: 150.03 ± 67.96 mg.dL^-1^, BG: 192.60 ± 66.59 mg.dL^-1^, HbA1c: 8.92 ± 0.84%) and serum C-peptide levels below the normal range (1.39 ± 1.12 ng.mL^-1^), which characterize pancreatic beta cell failure.

**Table 1:** Mean, median and standard deviation of the main variables at the beginning of the study (C3)

Factor	N	Mean	Median	SD	Min	Max
C3						
FG (mg.dL^-1^)	40	150.03	137.50	67.96	45.00	341.00
BG (mg.dL^-1^)	40	192.60	201.00	66.59	72.00	332.00
HbA1c (%)	40	8.92	9.10	0.84	7.5	7.50
C-peptide (ng.mL^-1^)	40	1.39	1.15	1.12	0.10	4.20

BG: bedtime glucose; FG: fasting glucose; SD: standard deviation – Wilcoxon Test.

Data from the descriptive analysis of the demographic characteristics obtained at consult C7 before randomization, after treatment with NPH insulin to obtain adequate glycemic control and after homogenization of the group (phase 1) are presented in Table 2, discriminated by gender. The age of the population studied ranged from 39 to 60 years (female: 52.9 ± 6.55 years; male: 49.45 ± 6.30 years) with no participation of elderly individuals. The majority of patients were overweight, with females presenting BMIs ranging from 24.79 to 33.78 kg.m^-2 ^(median: 27.16 kg.m^-2 ^and mean: 28.34 ± 2.84 kg.m^-2^) and males presenting BMIs ranging from 20.02 to 34.68 kg.m^-2^ (median: 27 kg.m^-2^ and mean: 27.7 ± 4.37 kg.m^-2^). The mean time living with a diabetes diagnosis was over 10 years (145 ± 73.65 months for females and 130.20 ± 74.21 months for males); for glycemic control, females presented the following clinical parameters: HbA1c–8.14 ± 0.86%, FG–144.45 ± 61.24 mg.dL^-1^ and BG–96.75 ± 35.03 mg.dL^-1^; and males presented the following clinical parameters: Hba1c–7.95 ± 0.63%, FG–93.20 ± 26.71 mg.dL^-1^ and BG–131.5 ± 45.16 mg.dL^-1^. The mean and median C-peptide (CP) values were lower than the normal range in both females (mean: 1.05 ± 0.91 ng.mL^-1^; median: 0.75 ng.mL^-1^) and males (mean: 0.92 ± 0.67 ng.mL^-1^; median: 0.80 ng.mL^-1^). Renal function was assessed by measuring serum creatinine, which was within the normal range for gender and age in all patients (females: maximum value: 1.11 mg.dL^-1^; minimum value: 0.44 mg.dL^-1^; mean: 0.82 ± 0.19 mg.dL^-1^; median: 0.84 mg.dL^-1^ and males: maximum value: 1.27 mg.dL^-1^; minimum value: 0.56 mg.dL^-1^; mean: 0.92 ± 0.18 mg.dL^-1^; median: 0.89 mg.dL^-1^). The antidiabetic drugs allowed at the beginning of the study were metformin at a mean dose of 1466.25 ± 608.51 mg for females and 1657.50 ± 583.38 mg for males and NPH insulin at a daily dose of 0.74 ± 0.27 IU.kg (insulin units per kilogram of body weight) for females and 0.63 ± 0.19 IU.kg for males.

**Table 2:** Descriptive analysis of the demographic characteristics by gender in each variable evaluated at the end of phase 1 (C7, pre-randomization)

Females			

Variable	Mean	Median	Standard deviation
Age (years)	52.90	54.50	6.55
Weight (kg)	71.23	70.10	7.11
Height (m)	1.59	1.60	0.05
BMI (kg.m^-2^)	28.34	27.16	2.84
Time living with the diabetes diagnosis (months)	145.20	138.00	73.65
BG (mg.mL^-1^)	144.45	142.50	61.24
FG (mg.mL^-1^)	96.75	88.50	35.03
HbA1c (%)	8.14	8.15	0.86
C-peptide (ng.mL^-1^)	1.05	0.75	0.91
Creatinine (mg.dL^-1^)	0.82	0.84	0.19
NPH dose (IU.kg^-1^)	0.74	0.74	0.27
Metformin (mg)	1466.25	1700.00	608.51

**Males**			

**Variable**	**Mean**	**Median**	**Standard deviation**

Age (years)	49.45	51	6.30
Weight (kg)	79.74	74.05	15.69
Height (m)	1.69	1.69	0.06
BMI (kg.m^-2^)	27.70	27.00	4.37
Time living with the diabetes diagnosis (months)	130.20	108.00	74.21
BG (mg.mL^-1^)	131.15	116.50	45.16
FG (mg.mL^-1^)	93.20	92.00	26.71
HbA1c (%)	7.95	7.85	0.63
C-peptide (ng.mL^-1^)	0.92	0.80	0.67
Creatinine (mg.dL^-1^)	0.92	0.89	0.18
NPH dose (IU.kg^-1^)	0.63	0.61	0.19
Metformin (mg)	1657.50	1700.00	583.38

The comparison between the main variables (HbA1c, FG and BG) obtained at the beginning (C3) and end (C7) of phase 1 (before randomization), during which the patients received NPH insulin Humulin aiming for better glycemic control, is displayed in Table 3. A significant reduction (p < 0.01) in Hba1c (8.92 ± 0.84% *vs*. 8.04 ± 0.75%), FG (150.03 ± 67.96 mg.dL^-1^*vs*. 94.98 ± 30.80 mg.dL^-1^), BG (192.60 ± 66.59 mg.dL^-1^*vs*. 137.8 ± 53.54 mg.dL^-1^) and C-peptide (1.39 ± 1.12 ng.mL^-1^*vs*. 0.99 ± 0.74 ng.mL^-1^) were observed.

**Table 3:** Comparison of the glycemic profile of the general group before and after the consults in phase 1 (run in); C3 vs. C7

	Consults		p
	C3	C7	
HbA1c (%)	8.92 (± 0.84)	8.04 (± 0.75)	< 0.01
FG (mg/dL)	150.0 (± 67.96)	95 (± 30.80)	< 0.01
BG (mg/dL)	192.6 (± 66.6)	137.8 (± 53.54)	< 0.01
C-peptide (ng/mL)	1.39 (± 1.12)	0.99 (± 0.74)	< 0.01

BG: bedtime glucose; SD: standard deviation – Wilcoxon Test; FG: fasting glucose.

Table 4 presents the main descriptive characteristics of the study population after randomization (C8). The comparison of the different variables between the Gansulin and Humulin groups revealed that the primary variables were similar between the groups (HbA1c: 8.18 *vs*. 7.91%, p = 0.24; FG: 94.50 *vs*. 95.45 mg.dL^-1^, p = 0.92; BG: 130.35 *vs*. 145.25 mg.dL^-1^, p = 0.39), as were the secondary variables (BMI: 29.01 *vs*. 27.03 kg.m^-2^, p = 0.09; C-peptide: 0.98 *vs*. 1.00 ng.mL^-1^, p = 0.94; creatinine: 0.83 *vs*. 0.92 mg.dL^-1^, p = 0.11; insulin dose: 0.75 *vs*. 0.62 IU.kg^-1^, p = 0.07; metformin dose: 1636 *vs*. 1487 mg, p = 0.44; time living with the diabetes diagnosis: 138 *vs*. 137.4 months, p = 0.98), except for the variable age, for which the individuals from the Gansulin group were significantly younger (48.7 *vs*. 53.65 years, p = 0.01).

**Table 4:** Comparison of the different variables between the two insulin groups, obtained after phase 1 (run in) (C8)

Insulin	HbA1c (%)	FG (mg.mL^-1^)	BG (mg.mL^-1^)	Age (years)	Weight (kg)	Height (m)
Gansulin	8.18	94.50	130.35	48.70	78.06	1.64
Humulin	7.91	95.45	145.25	53.65	72.90	1.64
p	0.2410	0.9257	0.3906	0.0128	0.1876	0.9310

**Insulin**	**BMI (kg.m^-2^)**	**C-peptide (ng.mL^-1^)**	**Creatinine (mg.dL^-1^)**	**Insulin Dose (IU.kg^-1^)**	**Metformin (mg)**	**Time living with the diabetes diagnosis (months)**

Gansulin	29.01	0.98	0.83	0.75	1636.25	138.00
Humulin	27.03	1.00	0.92	0.62	1487.50	137.40
p	0.0921	0.9444	0.1163	0.0695	0.4418	0.9796

HbA1c (%); FG: fasting glucose (mg.mL^-1^); BG: bedtime glucose (mg.mL^-1^); weight (kg); BMI (kg.m^-2^); C-peptide (ng.mL^-1^); creatinine (mg.dL^-1^); insulin dose (IU.kg^-1^); metformin (mg). Test: Tukey-Kramer, using the means adjusted by the least squares method.

Table 5 demonstrates that both insulin formulations were effective at reducing HbA1c levels (Humulin: 7.91 *vs*. 6.56%, p = 0.001; Gansulin: 8.18 *vs*. 6.65%, p = 0.001) but without significant changes in FG levels (Humulin: 95.45 *vs*. 85.63 mg.dL^-1^, p = 0.456; Gansulin: 94.50 *vs*. 110.29 mg.dL^-1^, p = 0.147) and BG levels (Humulin: 145.25 *vs*. 120.02 mg.dL^-1^, p = 0.195; Gansulin: 130.35 *vs*. 117.47 mg.dL^-1^, p = 0.422).

**Table 5:** Comparison of the glucose profiles obtained with the two insulin formulations between the first consult after randomization (C8) and the final consult (C21)

Glycemic profile	C8	C21	p

Humulin			
FG	95.45	85.63	0.456
HbA1c	7.91	6.56	0.001
BG	145.25	120.02	0.191

**Gansulin**			

FG	94.50	110.29	0.147
HbA1c	8.18	6.65	0.001
BG	130.35	117.47	0.422

BG: bedtime glucose; FG: fasting glucose; Wilcoxon Test.

As shown in Table 6, at the end of the study (end point), there was no significant difference in the primary variables between the Gansulin and Humulin groups: HbA1c (6.65 *vs*. 6.56% p = 0.65), FG (110.29 *vs*. 85.63 mg.dL^-1^, p = 0.12), and BG (117.47 *vs*. 120.02 mg.dL^-1^, p = 0.87), respectively. Similarly, there were no significant difference in the secondary variables between the groups: BMI (28.01 *vs*. 27.89 kg.m^-2^, p = 0.60), C-peptide (1.54 *vs*. 1.01 ng.mL^-1^, p = 0.13), creatinine (0.85 *vs*. 0.83 mg.dL^-1^, p = 0.60), and insulin dose (0.74 *vs*. 0.68 IU.kg^-1^, p = 0.38), with the exception of metformin, with the Gansulin group requiring larger doses of this drug (1787 *vs*. 1487 mg, p = 0.029).

**Table 6:** Comparison of means (end point) adjusted by the least squares method after an analysis of covariance with a baseline correction for the different variables

Insulin	Means				
	HbA1c (%)	FG (mg.mL^-1^)	BG (mg.mL^-1^)	Weight (kg)	BMI (kg.m-^2^)
Gansulin	6.65	110.29	117.47	75.42	28.018
Humulin	6.56	85.63	120.02	75.24	27.89
p	0.6536	0.1207	0.8734	0.7576	0.6056

**Insulin**	**C-peptide (ng.mL-1)**	**Creatinine (mg.dL^-1^)**	**Insulin dose (IU.kg-1)**	**Metformin (mg)**	

Gansulin	1.54	0.85	0.74	1787.26	
Humulin	1.01	0.83	0.68	1486.80	
p	0.1284	0.6051	0.3826	0.0298	

HbA1c (%); FG: fasting glucose (mg.mL^-1^); BG: bedtime glucose (mg.mL^-1^); weight (kg), BMI (kg.m^-2^), C-peptide (ng.mL^-1^); creatinine (mg.dL^-1^); insulin dose (IU.kg^-1^); metformin (mg).

Test: Tukey-Kramer, using the means adjusted by the least squares method.

Sixty-six hypoglycemic events were observed during this study, with no significant difference between the two insulin formulations: 36 events were observed in the Gansulin group, and 30 events were observed in the Humulin group. No severe hypoglycemic episode was observed during the study.

## DISCUSSION

To the best of our knowledge, this is the first randomized controlled trial comparing the efficacy and safety of a new brand of NPH insulin, Gansulin, which is not available in Brazil, with Humulin, which is an NPH insulin that has been available in Brazil for decades. A Chinese study conducted by Quin and cols. (13) was the only study similar to ours that is available in the specialized medical literature. In that study, the authors compared the clinical efficacy, safety and cost between the insulin formulations Gansulin and glargine in 200 patients with T2DM who were randomly divided into two groups of 100 patients and treated for three months. The researchers found no significant difference in efficacy (HbA1c and FG) and safety (hypoglycemia), but the costs with glargine were significantly higher than those with Gansulin.

Our data demonstrate that after phase 1, in which all patients were treated with NPH insulin Humulin and then randomized into two groups, only the variable age was significantly different (p = 0.012), which raises questions about the inclusion of patients with latent autoimmune diabetes in adults (LADA) in group 1; however, this possibility is unlikely because all patients included in the study were anti-GAD-negative (14,15). Another factor to be considered relative to the variable age is that older patients usually have decreased renal function, which can affect clinical kinetics and response. In addition, treatment adherence, ease of handling insulin and physical activity may also be affected by age and thereby may affect the results. We believe, however, that these factors most likely did not affect our data because, although the groups were not matched for age, the study protocol excluded elderly patients (> 60 years); therefore, an age-related reduction in renal function was unlikely. Additionally, serum creatinine levels were similar in both groups. Furthermore, insulin vials were checked weekly to assess treatment adherence, and all patients systematically received instructions for both insulin administration and glucose monitoring by the nutritionist involved in the study, who received training in Diabetes Education by the Brazilian Society of Diabetes (*Sociedade Brasileira de Diabetes* – SBD) and the Juvenile Diabetes Association (*Associação de Diabetes Juvenil* – ADJ).

At the end of phase 1 of the study, there was already a significant decrease in the levels of glycated hemoglobin, FG and BG without a significant difference between the two groups at the time of randomization (Tables 2 and 3), demonstrating the efficacy of this treatment phase in the homogenization of the groups. A significant reduction in serum C-peptide levels between consults 3 and 7 was observed, which possibly reflects decreased insulin resistance due to a reduction in glucose toxicity. The low levels of C-peptide in our population demonstrates the failure of pancreatic beta cells in patients living with a diabetes diagnosis for over 10 years and confirms the concept that T2DM patients often require the use of exogenous insulin to achieve glycemic control (7,16). A study conducted by Holman (17) demonstrated that a reduction in beta cell function begins between 10 and 12 years before the diabetes diagnosis, and no antidiabetic drug used during the study was able to prevent or slow the progression of this phenomenon. These data are similar to the data found by Bagust and Beale (18), which influenced the major international societies to introduce therapeutic insulin regimens into their guidelines, as we did our study.

Starting at consult 7, both insulin formulations were able to maintain the reduction in glycated hemoglobin levels until their complete normalization, as shown in Table 5. At the end of the study (Table 6), both insulin formulations were equally effective in achieving the glycemic control assessed by the primary variables (HbA1c, FG and BG), and similar results were observed for the secondary variables, except for the daily dose of metformin, with the Gansulin group requiring higher amounts of this drug. This finding would suggest a greater potency of Humulin insulin; however, the insulin requirements were identical in both groups, which may call into question the clinical relevance of this finding. Another pertinent observation was the fact that although the mean body weight was not significantly different between the two groups at both the initial (C3) and final (C21) consults, there was a weight reduction of 3.18 kg in the Gansulin group, while in the Humulin group, a gain of 2.32 kg was observed. Because the present investigation was a short-term study with weekly consults and nutritional orientation from a professional, these factors might have been important motivational factors that could explain the weight loss; however, the discrepancy between the two groups might be partially explained by the higher dose of metformin, which is known to promote weight loss, in the Gansulin group.

Our results are similar to those found by other groups for the glycemic control achieved with the introduction of NPH insulin in hyperglycemic patients using oral antidiabetic drugs (7).

Regarding safety, which was measured by the number and intensity of hypoglycemic events, no significant difference was observed between the two insulin formulations.

The main limitations of our study were as follows: (1) the small number of patients analyzed and (2) the short time of the treatment. Therefore, we suggest that future studies should be performed involving larger numbers of patients treated for longer periods and including an analysis of the cardiovascular safety and risk of malignant tumors.

The present study suggests similar efficacy profiles between the NPH insulin formulations Humulin and Gansulin in the treatment of patients with T2DM, which therefore indicates the possibility of introducing a new commercial form of human NPH insulin in addition to the preparations already used in public and private systems, with a possible reduction in costs.

In conclusion, several randomized clinical trials have demonstrated the importance of proper glycemic control (HbA1c < 7.0%) in patients with T2DM to prevent the microvascular complications associated with this disease. These and other clinical trials have also shown that the natural history of diabetes involves a progressive loss of beta cell function that consequently requires the use of multiple drugs to treat these patients, including insulin. The high prevalence of type 2 diabetes mellitus in the Brazilian population, similar to what occurs in other nations, has burdened the budget of private and public health care systems, which often leads to inadequate treatment and control. Therefore, the implementation of measures to reduce these costs without jeopardizing the quality of care for patients with diabetes is imperative. The availability of NPH insulin with an efficacy and safety similar to the gold standard but with a lower price can be one of the strategies to reduce financial costs.
